# Processing Cycle Efficiency to Monitor the Performance of an Intelligent Tube Preparation System for Phlebotomy Services

**DOI:** 10.3390/ijerph18179386

**Published:** 2021-09-06

**Authors:** Ming-Feng Wu, Jen-Ying Li, Yu-Hsuan Lin, Wei-Chang Huang, Chi-Chih He, Jiunn-Min Wang

**Affiliations:** 1 Division of Chest Medicine, Department of Internal Medicine, Taichung Veterans General Hospital, Taichung 407, Taiwan; heriknoha@vghtc.gov.tw (M.-F.W.); huangweichangtw@gmail.com (W.-C.H.); 2 Department of Medical Laboratory Science and Biotechnology, Central Taiwan University of Science and Technology, Taichung 406, Taiwan; 3 Department of Pathology & Laboratory Medicine, Taichung Veterans General Hospital, Taichung 407, Taiwan; jenying@vghtc.gov.tw (J.-Y.L.); n1921223@vghtc.gov.tw (Y.-H.L.); 4 Department of Medical Technology, Jen-Teh Junior College of Medicine, Nursing and Management, Miaoli 350, Taiwan; 5 School of Medicine, Chung Shan Medical University, Taichung 402, Taiwan; 6 Ph.D. Program in Translational Medicine, National Chung Hsing University, Taichung 402, Taiwan; 7 Master Program for Health Administration, Department of Industrial Engineering and Enterprise Information, Tunghai University, Taichung 407, Taiwan; 8 College of Medicine, National Chung Hsing University, Taichung 402, Taiwan; 9 Department of Neurological Institute, Taichung Veterans General Hospital, Taichung 407, Taiwan

**Keywords:** waiting time, phlebotomy, process cycle efficiency, intelligent tube preparation system

## Abstract

Background: The waiting time (WT) for a phlebotomy is directly related to patient satisfaction with a health service. However, the processing time varies widely depending on the type of patients. Monitoring of the WT alone may not enable an effective evaluation of the lean performance of the medical staff for patients with different characteristics. The objective of this study was to use process cycle efficiency (PCE) to assess the performance of an intelligent tube preparation system (ITPS) which automatically labeled test tubes and conducted patient rerouting for phlebotomy services, and to interpret the WT during peak hours. Methods: Three time periods were used. The baseline period was from 1 July to 31 July 2014. Phase 1 was after the establishment of the ITPS, with patients ≥80 years old being rerouted. In phase 2, patients ≥78 years old were rerouted. Those data were recorded with a calling system and ITPS, respectively. Results: PCE was significantly improved from 12.9% at baseline to 51.1% (*p* < 0.001) in phase 1 and 53.0% (*p* < 0.001) in phase 2. The WT of 16.9 min at baseline was reduced to 3.8 min in phase 1 (*p* < 0.001), and 3.6 min in phase 2 (*p* < 0.001). Moreover, the results showed that a WT < 10 min was consistent with a PCE ≥ 25%. Conclusions: Establishing an ITPS for phlebotomy can significantly increase PCE and shorten the WT. Furthermore, the PCE ≥ 25% could be a good assessment reference for the management of appropriate human resources for phlebotomy services, although it is a complex parameter.

## 1. Introduction

A phlebotomy area is where samples of patients’ blood are collected and tested in a hospital to assess their health. These tests are related to the diagnosis or follow-up of patients with many types of disorders, and many different patients can require a blood test at any given time. However, the number of patients waiting for a blood test often varies significantly at different times throughout the day and on different days of the week [1]. As a result, it is difficult to control the waiting time (WT). Many previous studies have reported that a long WT is associated with a decrease in patient satisfaction with the overall quality of service, and the patient’s anxiety and distress [2,3,4,5]. Therefore, reduction in the WT for a phlebotomy is an essential issue for clinical health management.

The WT for a phlebotomy is related to the number of patients and the number of phlebotomists, the patient flow through the service, and the service process. Hammond et al. used queuing theory to determine the optimal number of phlebotomists required to provide a blood drawing service [6]. A previous study used staffing levels and the number of patients requiring a blood test to derive the estimated capacity, and reported that the WT of less than 10 min for 88% of patients increased to 100% after changes [4]. In addition, Jeon et al. reported an active-phlebotomist phlebotomy system in which a phlebotomist went directly to patients, which significantly decreased waiting times [1]. Furthermore, Woo et al. developed a real-time computer simulation program which effectively decreased the help time for phlebotomists and outpatients’ WT for a phlebotomy [7].

Management strategies have been shown to shorten the WT for a phlebotomy. It was reported that there was a 19% decrease in WT after changes were made to the collection of materials, the LabTracker automated database system was improved with wait time calculators and real-time information regarding patient status, and lower-complexity appointments were streamlined [8]. Gupta et al. also concluded that analyzing the feedback of root causes was effective at maintaining and improving phlebotomy services [9]. Several studies have used Lean Six Sigma with DMAIC (define, measure, analyze, improve, and control) to identify potential factors affecting a phlebotomist’s daily routine, and reported improvements in the WT [10,11]. These studies enhanced the performance of phlebotomists with regard to WT.

There was no standard reference for waiting times for phlebotomy services; however, it was generally accepted that a wait time of less than 10 min was good [4]; a WT shorter than 10 min was positively correlated with overall satisfaction. Hence, both increasing the performance of phlebotomists as discussed in the previous paragraphs and enrolling more staff are required to shorten waiting times. It should be noted that older patients often walk slowly, have a shorter step length, and can find it hard to maintain postural stability, while those in a wheelchair need more pathway space to the examination table and can require transfer assistance; therefore, the time required to draw blood from elderly or disabled patients may be longer than for general adults, and this may increase the overall WT [12,13,14]. As a result, only measuring the WT can ignore the effort required by the phlebotomist to perform their tasks.

Identifying an objective lean process indicator for phlebotomy is important. Process cycle efficiency (PCE) is a lean measurement of the amount of value-added time in a process [15,16]. It can provide a reasonable reference value for necessary value-added activities and non-value-added activities for a specific flow chain. A PCE value ≥ 25% indicates that the process has a considerably lean flow. However, there has been no study to address the threshold of 25% PCE for monitoring the performance of phlebotomies.

We installed an intelligent tube preparation system (ITPS) at our hospital in October 2018 [17]. The system automatically provides labeled test tubes and a rerouting service based on the patients’ features, including whether they are in wheelchairs, are elderly, or are part of the general population. The study aimed to use PCE to evaluate the performance of ITPS for service numbers and service time, and to interpret the WT during peak hours. Additionally, the WTs among those in wheelchairs, the elderly, and the general population were also compared as we use a PCE ≥ 25% to adjust for the elderly first policy.

## 2. Materials and Methods

### 2.1. Blood Drawing Counters at Baseline

**Figure 1:**
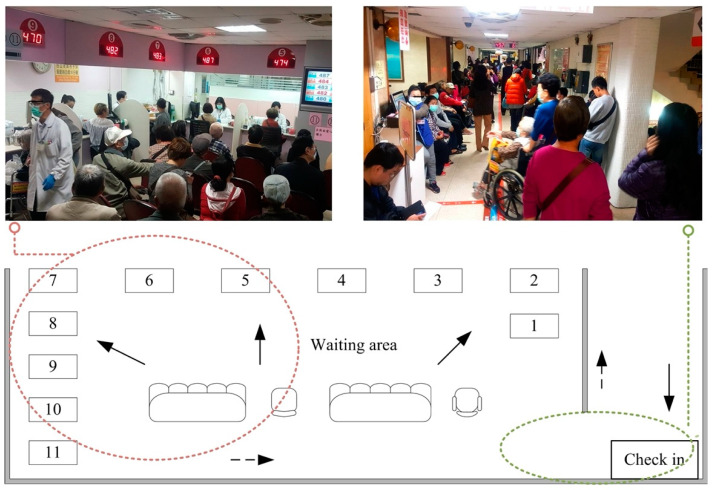
The phlebotomy service area and patient flow. Blood drawing counters 1–9 were for general subjects, and counters 10 and 11 were for wheelchair users at baseline. The area of the waiting area was about 49.9 m^2^. Solid arrow: the subjects arriving; Dotted arrow: the subjects leaving.

Data collected between 1 July and 31 July 2014 was defined as the baseline period. There were a total of 11 blood drawing counters during this period, of which two were priority counters for disabled patients with wheelchairs. After the patients checked in through the calling system, they rested in the waiting area. Once the phlebotomist called a patient’s number, their tubes were labeled manually (Figure 1).

### 2.2. The Establishment of ITPS

**Figure 2:**
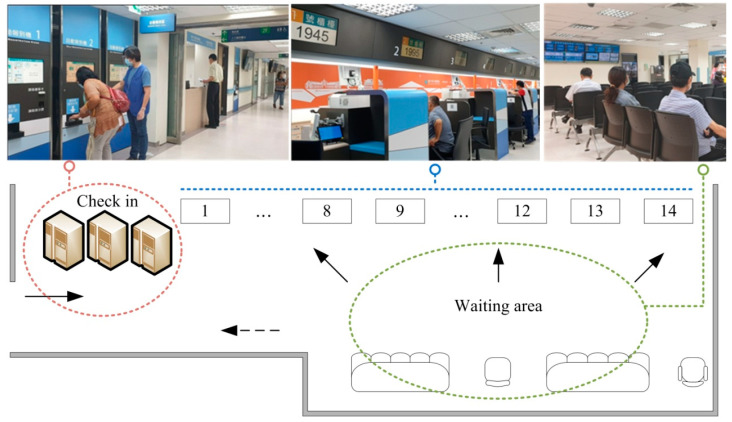
The phlebotomy service area and patient flow. Blood drawing counters 1–12 were for general subjects and 1–8 were priority counters for elderly subjects, while counters 13 and 14 were for wheelchair users. Age ≥80 years was defined as elderly from 1 November 2018 (phase 1), while age ≥78 years was defined as elderly from 1 July 2019 (phase 2). The area of the waiting area was about 62.4 m^2^. Solid arrow: the subjects arriving; Dotted arrow: the subjects leaving.

There were 14 blood drawing counters after ITPS was installed on 31 October 2018, of which two were for wheelchair users and eight were priority counters for elderly patients. The ITPS had a storage tank for different types of test tubes and was controlled by a computer. Patients received their labeled tubes immediately when they checked in with ITPS (Figure 2).

### 2.3. Blood Drawing Counters after the Establishment of ITPS

The ITPS allocated the elderly first at eight of the counters, and subjects ≥80 years old were given a high priority service between 1 November 2018 and 31 May 2019 (defined as phase 1). This was then adjusted to ≥78 years old between 1 June and 30 October 2019 (defined as phase 2) once a PCE much greater than 25% was met in phase 1. The rerouted principles were set as an algorithm embedded in ITPS.

### 2.4. Satisfaction Survey

A satisfaction survey was used to assess the whole blood drawing service. The survey machine was set on the blood drawing table. The patient voluntarily pressed the counter to select satisfied, neutral, or dissatisfied when they were finished with the service.

### 2.5. Data Analysis

The service number, staff number, waiting time (WT), and service time were recorded by a calling system in phase 1, while those were recorded by ITPS in phase 1 and phase 2. A value stream map (VSM) was used to indicate the process of phlebotomy. PCE, WT, and the ratio of service number to staff number (RSS) per hour were calculated. PCE was defined as value-added time (VAT) divided by the total lead time (TLT), where drawing blood was regarded as a value-added activity and WT was a non-value-added activity. All values were calculated monthly. Performance during peak times (7 am to 11 am) was analyzed among the three study periods with one-way analysis of variance (ANOVA) as the variables were normal and had displayed homogeneity of variances [18,19]. Otherwise, the Kruskal–Wallis test was used to conduct the analysis. The post-hoc test for the statistical significance was performed with multiplicity correction. In addition, an Independent *t*-test or Mann–Whitney U was used to compare the performance of the patients’ types (general population, wheelchair users, and the elderly) between phase 1 and phase 2 depending on whether there was normality or not. All statistical analyses were performed using Predictive Analysis Software version 18.0 (SPSS Inc., Chicago, IL, USA). Significance was set at *p* < 0.05.

## 3. Results

There were 1027, 1570, and 1453 services per day in the baseline, phase 1, and phase 2 periods, respectively (Table 1(A)), including 654 (63.7%), 1002 (64.1%), and 933 (64.2%) during the peak times. The RSS for the peak times was 14, 18, and 17 in the three study phases, respectively. The service number per staff in phase 1 and 2 was higher than that in the baseline period. However, there was no significant difference among them. In addition, the average service time was 153.7 s in phase 2. It was shorter than phase 1 with 161.7 s. This may due to the contribution of the general population and the elderly (Table 1(B)). Nevertheless, there was no significant difference in the average service time between the two phases after the multiplicity correction. Regarding WT, it was 16.9 min at baseline, was reduced to 3.8 min in phase 1 (*p* < 0.001), and 3.6 min in phase 2 (*p* < 0.001). Both phase 1 and 2 were much shorter than the baseline period. Moreover, PCE was significantly improved from 12.9% at the baseline to 51.1% (*p* < 0.001) in phase 1 and 53.0% (*p* < 0.001) in phase 2. However, there were no significant differences in waiting time and PCE between phase 1 and phase 2 for general patients, wheelchair users, or the elderly.

**Figure 3:**
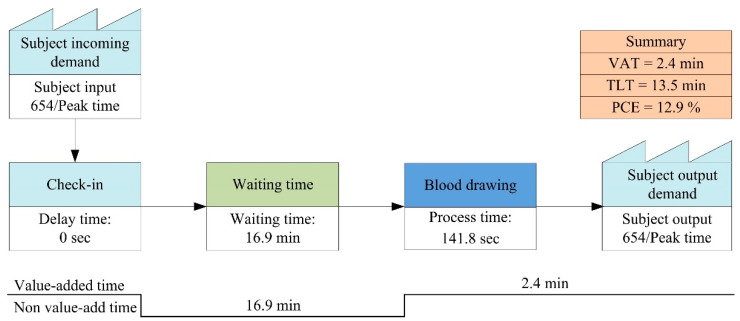
VSM of phlebotomy services: the baseline period.

**Figure 4:**
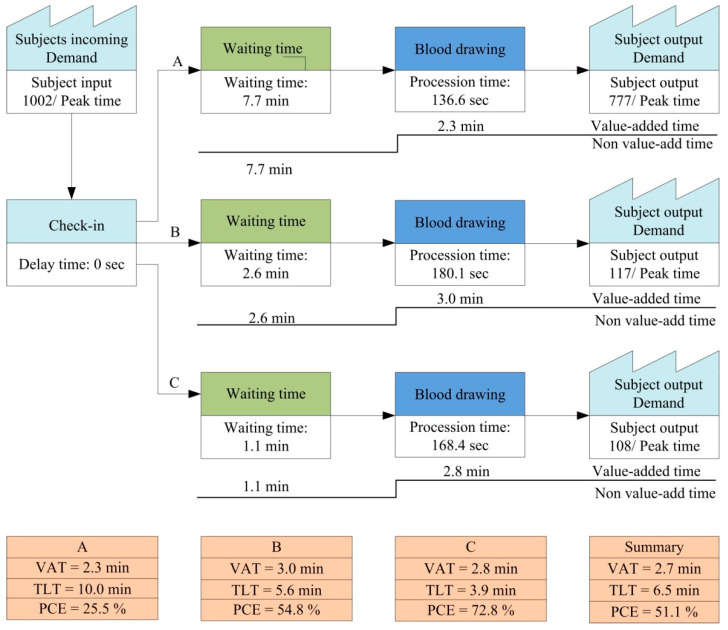
VSM of phlebotomy services: after establishing the ITPS (phase 2). A: general population; B: wheelchair users; C: elderly.

The VSM indicated an average of 654 patients per day during peak times in the baseline period (Figure 3). The VAT and TLT were 2.4 min and 13.5 min, respectively, and the waiting time (WT) and PCE were 16.9 min and 12.9%, respectively. In phase 1, there was an average of 1002 patients daily during peak times (Figure 4). After the ITPS had been installed, patient rerouting was performed based on the patient’s characteristics, i.e., general patients, wheelchair users, and the elderly. For these three subgroups, service time was 136.6 s, 180.1 s, and 168.4 s, respectively, while the waiting times were 7.7 min, 2.6 min, and 1.1 min. Accordingly, the PCE values were 25.5%, 54.8%, and 72.8%. The average VAT, TLT, and PCE were 2.7 min, 6.5 min, and 51.1%, respectively. The WT and PCE were better in phase 1 than during the baseline period.

The satisfaction survey revealed that the degree of satisfied patients was 85.00% at baseline (scoring from 34 subjects). The degree of satisfied patients (from the scoring population) for general patients, wheelchair users, and the elderly was 96.87% (*n* = 3064), 99.52% (*n* = 211), and 96.86% (*n* = 2071), respectively, for phase 1. The overall average of satisfied patients was 97.75%. The degree of satisfied individuals for general patients, wheelchair users, and the elderly was 98.43% (*n* = 3520), 97.89% (*n* = 521), and 98.78% (*n* = 1648), respectively, for phase 2. The results showed that the percentage of satisfied patients increased 12.75% from baseline to phase 1. Both the general population and the elderly had an improvement of 1.6% and 1.9%, respectively, from phase 1 to phase 2. However, the percentage of satisfied wheelchair users decreased by 1.6%.

**Figure 5:**
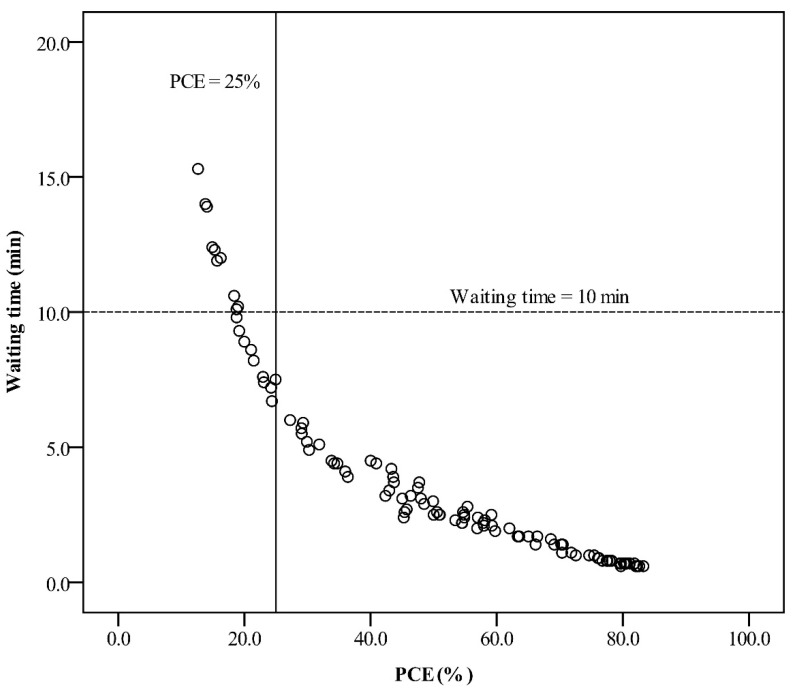
Plot of waiting time (minutes) against PCE (%) per month during phase 1. Vertical solid line: PCE of 25%; horizontal dashed line: waiting time of 10 min.

When WT was plotted against PCE, it showed an inverted relationship in phase 1 (Figure 5); a large PCE corresponded to a shorter WT. When the PCE was ≥25%, the waiting times were from 0.6 to 6.0 min (Table 2). However, once WT < 10 min, PCE went from 18.8% to 83.3%. The lean value was widely distributed. Additionally, the results also showed that there was up to 162 of the general population for phlebotomy services that experienced a WT less than 10 min with the PCE < 25%.

**Figure 6:**
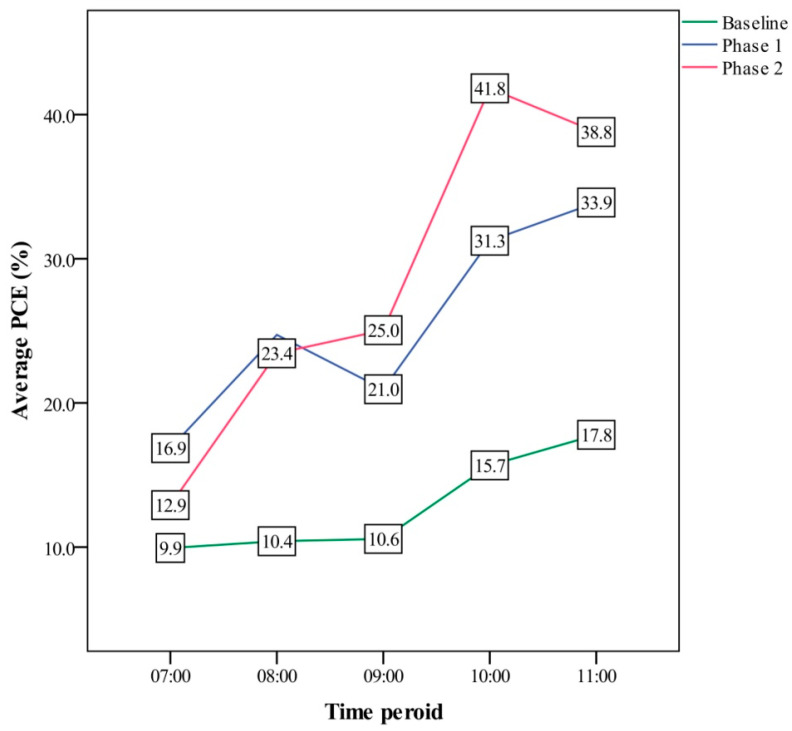
Comparison of PCE (%) at peak times among the three phases.

**Figure 7:**
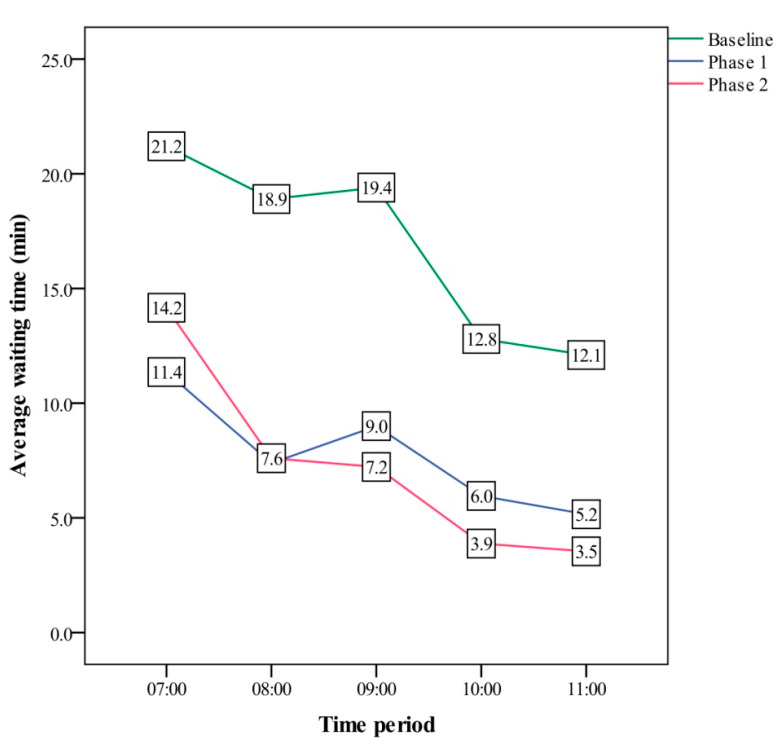
Comparison of waiting time (minutes) at peak times among the three phases.

Because the PCE for phlebotomy services on the elderly was much higher than 25% in phase 1, subjects ≥78 years old were included in the high priority service in phase 2 to try and improve this. Results showed that the waiting times were 7.3 min and 1.0 min, respectively. Moreover, the results revealed PCE values of 9.9% at 7 am and 17.8% at 11 am during the baseline period, 16.9% and 33.9% during phase 1, and 12.9% and 38.8% during phase 2 (Figure 6). There was a significant difference in PCE during peak times between the baseline and phase 1 (*p* < 0.001) and between the baseline and phase 2 (*p* < 0.001). With regards to the waiting times, there was a decrease from 21.2 min during the baseline period to 11.4 min during phase 2 at 07:00 a.m. and a decrease from 12.1 min at the baseline to 3.5 min in phase 2 at 11:00 a.m. (Figure 7). There was a significant difference in the WT during peak times between the baseline and phase 1 (*p* < 0.001) and between the baseline and phase 2 (*p* < 0.001). Both WT and PCE improved from baseline to phase 1 or phase 2. However, there was no significant difference in PCE (*p* = 1.000) or WT (*p* = 1.000) between phase 1 and 2 (Table 1(A)). Moreover, the results also showed that the PCE values for general patients, wheelchair users, and the elderly increased from phase 1 to phase 2, and they were 28.4%, 56.3%, and 74.2%, respectively, at phase 2 (Table 1(B)).

## 4. Discussion

The waiting time (WT) for a phlebotomy is related to patient satisfaction and the quality of service. After we installed an ITPS, the WT was reduced significantly from 16.9 min during the baseline period to 3.8 min in phase 1. The degree of satisfied patients rose by 12.75% from 85.00% to 97.75%. This was better than in a previous study where 94% of patients were satisfied with the phlebotomy services [9]. This significant improvement may have been due to the ITPS, which automatically provided labeled tubes when the subjects checked in instead of the staff labeling them manually when drawing blood. This change in procedure saved a large amount of time and allowed the staff to provide a faster phlebotomy service.

Many methods have been proposed to effectively improve phlebotomy services, including the effective use of human resources, information systems, and lean management, that we have reported in the introduction. Moreover, another study with the elimination of non–value-added steps and modifications to operational processes by increasing capacity to handle workload during peak hours led to a reduction in average WT from 21 to 5 min for phlebotomy services [20]. However, there is currently no consensus on a reasonable WT; whether the expenses required to shorten the WT, with regards to human resources and equipment, are worthwhile is currently under debate. In addition, phlebotomy staff may spend more time with wheelchair users and the elderly than with the general population, and this can also be improved by rerouting these patients according to their automatically labeled tubes. Our results in phase 1 showed that the time required to draw blood for wheelchair users, the elderly, and general patients was 180.1 s, 168.4 s, and 136.6 s, respectively. This supports the hypothesis that the processing times are different between different types of patients. The shorter average service time in phase 2 compared to phase 1 may be due to patients wearing more clothes in colder weather during a greater period of phase 1 (from 1 November 2018 and 31 May 2019) compared to phase 2 (between 1 June and 30 October). There was a significant difference for the general population and the elderly. This finding demonstrated that service time was a critical component of the lean value for phlebotomy services.

VSM is a method used to illustrate and analyze the lean value of a production process [21]. It has been used to ascertain and measure current and future efficiencies, allowing us to adapt to changes in healthcare as well as developments in emergency departments [21,22]. In contrast to WT, PCE is a lean indicator of the amount of value-added time in a process for VSM [23]. We considered both the processing time and the WT for different patient groups, and this approach may be valuable for assessing phlebotomy services. Routing services have been shown to be beneficial for certain populations [24]. However, few management tools are currently available to monitor this. In the present study, we found that the overall PCE during peak times was only 12.9% in the baseline period, however, it increased to 51.1% in phase 1. In addition, the PCE values of general patients, wheelchair users, and the elderly were 25.5%, 54.8%, and 72.8%, respectively, all of which showed a lean flow.

In particular, the elderly group had much higher PCE values of 25% in phase 1. Hence, subjects ≥78 years old were included in the elderly group in phase 2 and rerouted accordingly. The PCE values of general patients and the elderly were 28.4% and 74.2% in phase 2, respectively, and the waiting times were 7.3 min and 1.0 min, respectively. The degree of satisfied general patients increased from 96.87% in phase 1 to 98.43% in phase 2, while it increased from 96.86% to 98.78% for elderly patients. The increased improvement in PEC that was observed for general patients more so than for elderly patients was probably due to the lowering of the cut-off age for the elderly priority group to ≥78 years. This, therefore, reduced the number of general patients based on the PCE ≥ 25% of the elderly in phase 1. PCE was not used to interpret wheelchair users between the two phases of the study. Although PCE increased by 1.5%, the degree of satisfied wheelchair users decreased by 1.6%. The causes for this change should be considered, such as whether suitable sitting arrangements have been provided or whether other special assistance is required.

The 5S principle has been used to reduce the WT for phlebotomies [25]. The establishment of an ITPS significantly improved the WT and PCE in the current study and may meet the abundant components of the 5S principle. For example, the ITPS provided automatically labeled tubes when the patients checked in. This feature therefore reduced the processing time and avoided identification errors by staff, which conforms to the “Sort” and “Set” of the 5S approach. Moreover, the rerouting service based on the patients’ types could enable identification of service for “Shine” within the 5S approach. As a result, the RSS was 14 per hour in peak times. The RSS increased to 18 and 17 in phase 1 and phase 2, respectively. In addition, the space of the waiting room was 49.9 m^2^ at baseline, which increased to 62.4 m^2^ following the establishment of ITPS. This could decrease the staggering line from the waiting room to the service counter and could meet the “Set” of the 5S approach.

Many diseases are spread via droplets or aerosolized particles. Crowd gathering is a major risk factor for the transmission of these diseases. The 2019 coronavirus pandemic (COVID-19) was well known for this type of transmission [26,27,28]. A report concluded that COVID-19 will persist and become a recurrent seasonal disease [29]. It has been suggested that the maintenance of social distancing could reduce the spread of disease, however, the phlebotomy area is almost always crowded in a hospital, especially in peak times. The latest study proposed an artificial intelligence-based system to predict patient WT in the phlebotomy unit [30]. Subjects may have blood collection completed according to the predicted time to avoid gathering in the waiting area. However, a predicted error of ±2 min may cause the elderly or wheelchair users to miss the turn. The current study proved that the establishment of ITPS could decrease the number of people gathering by reducing the WT. Moreover, the study showed that all the WT was less than 10 min as PCE ≥ 25%. An evaluation of PCE will have the benefit of sustained monitoring of waiting times and could decrease crowd gathering and human resources in the face of an outbreak. In contrast, there were PCE < 25% as WT < 10 min. This was a fourfold (162 versus 40) increase in the general population with respect to PCE ≥ 25% for phlebotomy services. This was a case of a crowded situation. Monitoring WT alone for less than 10 min will miss the intervention of human resource management, such as increased staffing levels to decrease crowd gathering.

The general subjects accept a WT of 10 min. Nevertheless, the shorter the waiting time, the higher the degree of satisfaction. Therefore, there is no standard waiting value for adjusting the priority of different types of patients. The current study used PCE greater than 25% to reduce age as a priority strategy for the elderly. Results proved that not only was WT less than 10 min but also satisfaction was improved. In addition, to shorten the WT, more medical staff may have to be added. This current study also confirmed the service time required by different types of patients. The use of PCE may reasonably manage human resources. Once it exceeds 25%, it may reduce the number of staff. However, the optimal value of PCE for phlebotomy services was not determined in the current study. This can be determined in future studies when we put the PCE for phlebotomy management into practice.

ITPS can calculate the number of services, WT, and processing time, however, it cannot monitor the WT before subjects check in to the hospital. In addition, the initial collection of data in this study was from July 2014 only (baseline). This may have caused a bias in the analysis in this study. Different specifications of ITPS were set in many hospitals in Taiwan, Japan, Korea, etc. [17,31]. The study suggested that PCE will be a useful tool for the assessment of the lean process of services as adapted by ITPS.

## 5. Conclusions

An ITPS with automatically labeled tubes and a rerouting service for phlebotomies can significantly increase PCE and shorten the WT. Ultimately, the measurement of PCE values could be helpful in the management of human resources for health services.

## Figures and Tables

**Table 1:** The characteristic of the study: (**A**) Service details of phlebotomy among the baseline period (July 2014), phase 1 (1 November 2018 to 31 May 2019), and phase 2 (1 June and 30 October 2019); (**B**) The comparison of the subgroup between phase 1 and phase 2 in peak time.

**(A)**									
**Characteristic**	**Baseline**		**After the Establishment of the ITPS**						
			**Phase 1**		**Phase 2**		**F**		***p*-Value**
	Per Day	PT	Per Day	PT	Per Day	PT			
Total service number ^λ^	1027	654 (63.7%)	1570	1002 (64.1%)	1453	933 (64.2%)	-		0.220
Service number per hour ^λ^	64	131	101	200	92	187	-		0.063
RSS ^λ^	13	14	16	18	14	17	-		0.253
Service time (s) ^κ^ α (*p* = 0.185); β (*p* = 0.801); γ (*p* = 0.070)	167 ± 27.0	141.8 ± 9.4	146.0 ± 17.0	161.7 ± 22.7	147.8 ± 27.1	153.7 ± 24.1	3.866		0.023 *
Waiting time (min) ^κ^ α (*p* < 0.001 *); β (*p* < 0.001 *); γ (*p* = 1.000)	11.1 ± 6.1	16.9 ± 4.2	8.1 ± 5.3	3.8 ± 3.5	8.7 ± 6.4	3.6 ± 3.9	30.865		<0.001 *
PCE (%) ^λ^ α (*p* < 0.001 *); β (*p* < 0.001 *); γ (*p* = 1.000)	26.1 ± 19.4	12.9 ± 3.6	28.6 ± 13.4	51.1 ± 21.9	29.2 ± 15.1	53.0 ± 22.4	-		0.001 *
**(B)**									
**Characteristic**	**General Population**			**Wheelchair Users**			**The Elderly**		
	**Phase 1**	**Phase 2**	***p*-Value**	**Phase 1**	**Phase 2**	***p*-Value**	**Phase 1**	**Phase 2**	***p*-Value**
Service number per hour	155	141	0.504 ^ξ^	23	24	0.910 ^ξ^	22	22	0.314 ^ξ^
Service time (s)	136.6 ± 4.9	130.1 ± 8.0	0.001 *^,Φ^	180.1 ± 19.7	170.2 ± 25.4	0.110 ^Φ^	168.4 ± 10.7	160.8 ± 13.2	0.017 *^,Φ^
Waiting time (min)	7.7 ± 3.5	7.3 ± 4.5	0.635 ^Φ^	2.6 ± 0.9	2.5 ± 1.8	0.126 ^ξ^	1.1 ± 0.7	1.0 ± 0.7	0.109 ^ξ^
PCE (%)	25.5 ± 9.1	28.4 ± 13.4	0.328 ^Φ^	54.8 ± 9.0	56.3 ± 9.9	0.557 ^Φ^	72.8 ± 11.3	74.2 ± 12.4	0.239 ^ξ^

PT: peak time; RSS: the ratio of service number to staff number; ITPS: intelligent tube preparation system; ^κ^: ANOVA test; ^λ^: Kruskal–Wallis test; Multiplicity correction for ^κ^ and ^λ^ were Bonferroni and Dunnett, respectively. α, β and γ were the comparisons of baseline and phase 1, baseline and phase 2, as well as phase 1 and phase 2, respectively. ^Φ^: Independent *t*-test; ^ξ^: Mann–Whitney U. *: *p* < 0.001.

**Table 2:** The indicator comparison with PCE ≥ 25% and waiting time < 10 min.

	PCE ≥ 25%	WT < 10 Min	PCE < 25% When WT < 10 Min
Service number: general (per hour)	40 (8, 161)	53 (8, 178)	162 (114, 178)
Service number: wheelchair (per hour)	19 (8, 32)	24 (8, 38)	31 (10, 38)
Service number: aging (per hour)	15 (8, 28)	19 (8, 50)	26 (16, 50)
Service time (s)	167.7 (119.1, 217.3)	164.3 (119.1, 217.3)	135.9 (129.4, 149.5)
RSS	14 (11, 16)	15 (11, 21)	18 (15, 21)
Waiting time (min)	2.3 (0.6, 6.0)	2.9 (0.6, 9.8)	8.1 (6.7, 9.8)
PCE (%)	58.6 (27.2, 83.3)	54.8 (18.8, 83.3)	22.0 (18.8, 24.9)

## Data Availability

Those data were recorded with a calling system (from 1 July to 31 July 2014) and intelligent tube preparation system (from 1 November 2018 to 30 October 2019). The data meet the criteria for exemption from the Institutional Review Board (IRB) of Taichung Veterans General Hospital, Taiwan (Exemption No. CW21276A).
